# DMP: Detouring Using Multiple Paths against Jamming Attack for Ubiquitous Networking System

**DOI:** 10.3390/s100403626

**Published:** 2010-04-12

**Authors:** Mihui Kim, Kijoon Chae

**Affiliations:** 1 Department of Computer Science, North Carolina State University, 890 Oval Dr., Raleigh, NC 27614, USA; 2 Department of Computer Science and Engineering, Ewha Womans University, 11-1 Daehyun-dong, Seodaemun-gu, Seoul, 120-750, Korea; E-Mail: kjchae@ewha.ac.kr

**Keywords:** jamming attack, attack tolerant routing, multiple paths, ubiquitous networking system

## Abstract

To successfully realize the ubiquitous network environment including home automation or industrial control systems, it is important to be able to resist a jamming attack. This has recently been considered as an extremely threatening attack because it can collapse the entire network, despite the existence of basic security protocols such as encryption and authentication. In this paper, we present a method of jamming attack tolerant routing using multiple paths based on zones. The proposed scheme divides the network into zones, and manages the candidate forward nodes of neighbor zones. After detecting an attack, detour nodes decide zones for rerouting, and detour packets destined for victim nodes through forward nodes in the decided zones. Simulation results show that our scheme increases the PDR (Packet Delivery Ratio) and decreases the delay significantly in comparison with rerouting by a general routing protocol on sensor networks, AODV (*Ad hoc* On Demand Distance Vector), and a conventional JAM (Jammed Area Mapping) service with one reroute.

## 1. Introduction

**Figure 1.**
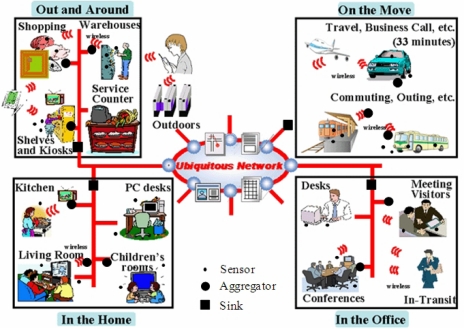
An example of a ubiquitous networking system.

*Ubiquitous computing* is used to refer to an information environment in which computers are installed everywhere, affecting all areas of a person’s life and operating autonomously in a network of linked computers to strongly support the modern human lifestyle. The *ubiquitous networking system* includes all the technologies needed for infrastructure that enables information to be exchanged anytime and anywhere through a high-speed, large-capacity, broadband network connecting homes, buildings and industrial systems, as shown in Figure 1 [1]. As one of the basic infrastructures, sensor networks provide much of the information needed to realize a convenient ubiquitous life. However, the proper security services are indispensable for actualizing the original goals of the ubiquitous networking system.

To date, research on security in the ubiquitous networking system has laid disproportionate emphasis on basic security mechanisms, such as authentication or key management. Due to the wireless characteristic or easy physical compromise of sensor nodes, these basic security services are indispensable. However, a defense against possible attacks is also essential to avoid negating much of the promise of ubiquitous networks, because attacks can still be performed even if network communication provides confidentiality and authenticity.

As one of the most threatening attacks on the ubiquitous networking system, the jamming attack can intentionally disrupt wireless transmission via interference, noise or collision at the receiver side. To launch the jamming attack, no special hardware is needed; the adversary simply listens to the open medium and broadcasts on the same frequency band as the network. It means that jamming is an effective, low cost attack from the point of view of an attacker, while it is very threatening to wireless users. It can occur either at the physical layer or access layer. Jamming attacks threaten the availability of network resources, and moreover permit real world damage to people’s health and safety exceeding simple damages such as loss of sensory data or energy exhaustion of nodes.

A.D. Wood *et al.* [2] presented basic defenses against these attacks such as spread-spectrum or authentication, but these straightforward defenses alone are not sufficient for protecting the availabilities of ubiquitous networks. In addition, utilization of the spread spectrum as a defense against jamming on the physical layer can be too energy-consuming to be widely deployed in resource-constrained sensors [3]. Moreover, representative sensor MAC (Media Access Control) protocols, such as S-MAC, B-MAC and T-MAC have considerable vulnerabilities to jamming attacks because of the feature of carrier sensing for transmission [4]. Thus, the simple solution of merely sleeping at the MAC layer after detection cannot be a fundamental solution [5]. Multipath routings on sensor networks [6,7] could be candidate solutions. However, though they set up multiple disjointed routes with the best hop, they do not provide immediate routes evading the jamming area. As an evasive method for smooth communication after detecting jamming, JAM (Jammed Area Mapping) simply focused on a mapping service of the jamming area [8]. Even though this is a meaningful partial solution, it cannot be a sufficient solution against jamming attacks because it takes time for the routing protocols to update the information, and a route that detours the determined jamming area may induce more jamming on the route if the amount of normal traffic passing the jamming area is huge. Thus, none of the existing defenses support the smooth transmission of normal traffic through immediate evasion of the jamming area.

In this paper, we design a routing method, called DMP (Detouring using Multiple Paths), for detouring the jamming (victim) area after detecting jamming and determining the jamming area. In the general case and in an area independent of the jamming attack, the method based on general sensor routing is performed. However, detour nodes at the boundary of the jamming area immediately detour normal traffic destined for the jamming area to forward nodes in neighbor zones. Multiple paths prevent flooding of the normal traffic on one route at the boundary of jamming area.

The rest of the paper is organized as follows. Section 2 discusses related work. Section 3 describes the detouring service using multiple paths. Section 4 presents our simulation evaluation of the proposed scheme, and finally Section 5 concludes this paper.

## 2. Related Work

As candidate solutions for evading jamming attacks, we analyze detour approaches on wired networks, general *ad-hoc* and sensor routings, and other evasive methods against jamming.

### 2.1. Detour Architectures on Wired Network

On wired networks, a flooding attack, one of the DDoS (Distributed Denial of Service) attacks, is a similar case to a jamming attack. In a flooding attack, distributed multiple agents consume some critical resources at the target server within the short time and deny the service to legitimate clients. As a side effect, they frequently create network congestion on the way from source to target, thus disrupting normal Internet operation and causing the connections of many users to be lost. However, because attack traffic generally overwhelms at a bottleneck toward the Internet or a target server, it is difficult to smoothly provided a detour service on wired networks. Thus, some researches [9,10] were proposed utilizing an overlay structure. SOS architecture using chord overlay [9] is geared toward supporting emergency services or similar types of communication and introduces randomness and anonymity into the forwarding architecture, making it difficult for an attacker to target nodes along the path to a specific SOS-protected destination. HOURS [10] using hierarchical overlays achieved DoS resilience in an open service hierarchy, such as a domain name server (DNS), lightweight directory access protocol (LDAP), or public key infrastructure (PKI). However, these overlay architectures are not appropriate for resource-constrained sensors, and detour services on wireless networks can be easily and quickly provided with simpler protocols than overlay protocols. In this paper, we design such a simple protocol for detour service against jamming attacks.

### 2.2. *Ad-Hoc*/Sensor Routing

The AODV (*Ad hoc* On Demand Distance Vector) protocol [11] is one of the most representative routing protocols designed for mobile *ad-hoc* networks, and also can be used on ubiquitous sensor networks. It is a reactive algorithm, meaning that it builds routes between nodes only as desired by source nodes. It maintains these routes as long as they are needed by the sources. However, after a route is set up, the route is not instantly adapted for faults or attacks on middle nodes. Such results will be shown in Section 4. Kang *et al.* [12] designed a routing protocol in mobile *ad hoc* networks in order to reduce control message overhead and maintain route paths, even where nodes move continuously at a high speed. It maintains continuously the path with low overhead through tracking the destination’s location, but the route is not quickly prepared for jamming on the middle nodes, like AODV.

Usually, most routings on ubiquitous sensor networks focus on energy efficiency in normal cases [6,7,13]. All these protocols provide the dynamics for changes of the network condition, but do not cope speedily with jamming conditions, because the new routes are regenerated between a sink and the sources. Specially, EAP [13] provides a long-lived sensor network through energy-aware routing protocol, but intermittent jamming induces instead considerable energy consumption for electing new cluster heads and configuring newly the network. In order to enhance the reliability of path and decrease the end-to-end delay, Tufail *et al.* [14] designed a routing protocol providing the reliable path through hotlines between gateway nodes, but this scheme also cannot defeat speedily the jamming around sensors.

On the other hand, multipath routings might become an effective countermeasure for the avoidance of jamming. However, mechanisms providing multiple paths simply focus on the energy efficiency as data centric routings [6,7] and data aggregation and in-network processing on hierarchical sensor networks [15], and moreover they do not provide the immediate avoidance of dynamic jamming areas.

### 2.3. Existing Defense Mechanisms against Jamming

A well-known attack on wireless communication, namely jamming, interferes with the radio frequencies of nodes. An adversary can easily disrupt the entire network with less than *N* jamming nodes on an *N*-size network. The standard defense against jamming involves various forms of spread-spectrum communication, but the main device used for ubiquitous networking, sensor nodes, will likely be limited to single-frequency use because of its low-cost, low-power character [5].

**Figure 2.**
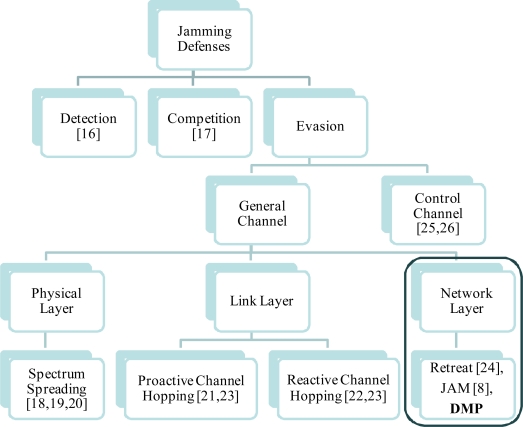
Existing jamming defenses and DMP.

Generally, research on jamming defenses is categorized as shown in Figure 2: *detection, competition, and evasion*. Detection using both PDR (Packet Delivery Ratio) and RSSI (Receive Strength Signal Indicator) on sensor networks is proposed [16], but the detection mechanism requires the following countermeasures until the jammer can be perfectly be eliminated. Competition approaches can be utilized with the control of transmission power or strong coding for error correction [17], but these methods are too energy-consuming to apply on sensor nodes.

Recently, evasion approaches have attracted considerable attention, because complete prevention against jamming is hard to implement and the provision of continuous service is important in the existence of jammers. These evasion approaches can be classified according to the affected layer: *physical layer*, *link layer*, and *network layer*. Spread spectrum has long been used to resist jamming attacks in wireless unicast environments. Chiang *et al.* [18,19] designed a scheme for broadcast jamming mitigation based on spread spectrum, especially DS (Directed Sequence) and FH-CDMA (Frequency Hopping-Code Division Multiple Access), and a balanced binary key tree and showed theoretical justifications of designed scheme. Specially, frequency hopping spread spectrum is based on the share of a secret spreading key (or code) between devices prior to the start of their communication. Strasser *et al.* [20] proposed establishment of jamming-resistant keys using uncoordinated frequency hopping based on the assumption that the attacker cannot jam all frequency channels on which the nodes communicate at the same. However, the utilization of spread spectrum as a defense against jamming on the physical layer can be too energy-consuming to be widely deployed in resource-constrained sensors.

As evasion approaches on link layer, channel hopping utilizes the fact that there are a number of orthogonal radio channels. For example, a representative sensor standard, IEEE 802.15.4 (e.g., CC2420 radio in MICAz motes) has 16 channels. Wood *et al.* [21] proposed a proactive (periodic) channel hopping scheme, coordinated synchronously. Reactive channel hopping, whereby radios switch channels to escape jamming, has been proposed to mitigate jamming in wireless sensor networks [22], and it occurs after radio jamming is detected and causes the entire network or only the jammed region to switch to a different radio channel networks. Khattab *et al.* [23] compared the proactive and reactive channel hopping schemes, and showed that reactive defense provides better jamming tolerance than a proactive one when considering communication availability. However, channel hopping is not sufficient for jamming defense, because the jammer can also change the jamming channel continuously and then it enlarges the channel switching overhead on nodes.

As one of the evasion methods on network layer, spatial retreat [24] is limited to mobile environments. As an evasive method used after detecting jamming, JAM (Jammed Area Mapping) simply focused on a mapping service for the jamming area [8]. JAM uses a priority message to inform the node’s neighbors of the attack detection, maps the jammed area as feedback for routing and reports to a base station for jamming localization. However, it takes time for the routing protocol to update the information, or for the base station to get the report and take follow-up measures. During this time, normal traffic routed to the jamming area may become congested or dropped. Moreover, the best single route to a destination generated by general routing protocols could easily become congested again. This may be worse, as traffic destined to a destination such as a sink could be greater or the jammed region could be bigger. Our simulation results will show these effects in Section 4.

On the other hand, researches on control channel jamming [25,26] have progressed, because jamming the control channel in wireless networks reduces the required power for performing a DoS attack by several orders of magnitude, and control channel jamming is particularly devastating for wireless *ad-hoc* networks due to their cooperative nature. Tague *et al.* [25] implemented multiple control channels over specified frequency bands and time slots, so that any subscriber can listen to them. Lazos *et al.* [26] proposed a randomized distributed scheme that allows nodes to establish a new control channel using frequency hopping in multi-channel ad-hoc networks. The protection of control channel utilizing multiple channels is important, but it is out of scope in this paper.

Thus, in this paper, we focus on a general evasion approach on network layer, independent of the specific characteristics on physical and link layer. Our scheme, DMP, provides immediate multiple paths for detouring normal traffic destined for the jamming area, and we will show that the immediate routes provide a higher PDS and lower delay than AODV or JAM with a route by various parameters. Table 1 is a summary of the most relevant related work.

## 3. Detouring Service via Multiple Paths

### 3.1. Basic Detouring Service

Our method of defense, DMP, focuses on achieving smooth communication directly after attack detection in the presence of a jammer. Thus, we assume the existence of detection and mapping mechanism for the victim area such as optimal detection policy against sophisticated jammer [3] and mapping service of the jamming area [8]. We will explain DMP with the following terms:

- *Victim/Jamming zone*: Zone including victim nodes against jamming attacks
- *Neighbor zone*: Adjacent zones of each zone
- *Destination zone*: Zone including a destination node
- *Detour node*: As the boundary node of the victim zone, it performs the algorithm shown in Table 2, thus it detours normal traffic with the determined multiple paths.
- *Forward node*: The first foothold nodes are used to detour normal traffic. After the detour nodes determine these nodes, they forward normal traffic to them.
- *Forward zone*: Zone determined by a detour node for detouring normal traffic

After mapping the victim zone and selecting the detour nodes, our method of defense provides new paths to the destination node. For efficiently detouring normal traffic, we divide the network into rectangular zones. We assume that each node knows its zone ID and neighbor zones, and can know the zone ID of a node through the node ID and calculate the distance between two zones. However, our method of defense can be adjusted to zones with other shapes such as a hexagonal zone or an arbitrary shaped zone. We will discuss the extension of this basic detouring service in subsection 3.2.

For our detouring service via multiple paths, each node has forward nodes included in each neighbor zone. When jamming attacks are detected, each detour node performs the algorithm shown in Table 2. If the next-hop of normal traffic is a node in the jamming area (line 1), it composes the set of forward nodes. In order to construct the proper set, it initially gathers the suitable forward zones among neighbor zones according to the distance from destination zone. If forward nodes in the neighbor zones are included in the set of jamming nodes, the zone is excluded from the set of forward zones (line 4). Moreover, if normal traffic has already been forwarded from a previous detour node, a zone including the previous node is excluded (***z*** ≠ ***dz***). Then, the forward nodes in the set of forward zones are sorted according to the distance of the included forward zone from the destination zone, and α nodes in the sorted list are chosen (line 10–13). Finally, the detour node detours normal traffic evenly to the determined forward nodes. However, if the next-hop of traffic is not included in the jamming area, the detour node simply routes the traffic according to the normal routing protocol.

**Figure 3.**
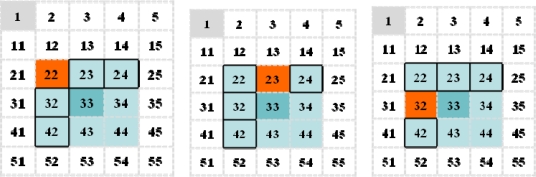
An example of forward zones according to victim zones (destination zone 1, victim zone is 22, 23 and 32 for each respective case.).

Figure 3 depicts an example of forward zones as victim zones. The nodes in zone 33 have neighbor zones 22, 23, 24, 32, 34, 42, 43 and 44. On general routing, the possible cases in which the next-hop of normal traffic is a node in the jamming area are as follows: jamming zone 22, 23 and 32, as shown in Figure 3. Thus, the forward zones determined by line 4 of the algorithm shown in Table 2 are zones, except for each jamming zone among 22, 23, 24, 32 and 42. For example, if each node has a forward node in each neighbor zone and **α** is equal to 2, then the detour nodes in zone 33 consist of two forward nodes in zone 23/32, zone 22/32 and zone 22/23 in the respective cases.

### 3.2. An Extension of a Routing Protocol RDSR [27]

**Figure 4.**
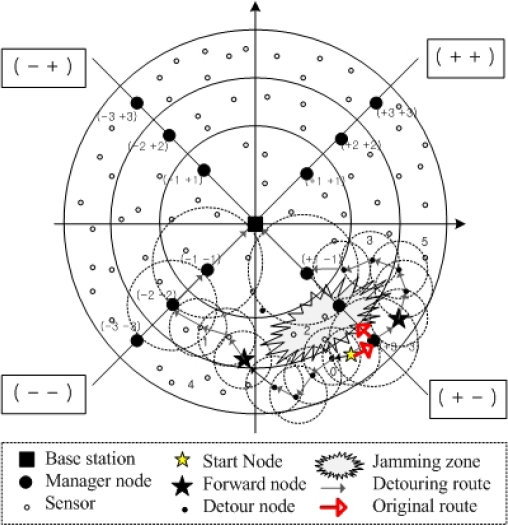
Rerouting example with extended RDSR in the jamming attack with α = 2.

DMP is based on the general routing protocols in the normal case or in any area except for the boundary of the jamming zone. Thus, we illustrate the extension of a sensor routing protocol, called Reactive Direction based Sensor Routing (RDSR), in order to reinforce the resistance against jamming with DMP. RDSR is one of the routing schemes considering the energy efficiency on sensor networks, as shown in Figure 4. We choose the RDSR with hierarchical topology, unlike the base DMP with rectangular zones. The assumed network consists of a base station, several manager nodes and lots of sensor nodes randomly distributed. The whole network is divided into four quadrants, namely (+ +), (+ −), (− +), and (− −), and then each quadrant is divided into sectors. Thus, each node tries to route the traffic toward the manager node if it exists within a 1-hop distance. Otherwise it finds the node with the smallest sector number as a next hop node or just selects a node with the same sector ID. If there is any detouring service in RDSR, the traffic from the start node is blocked at the (+3 −3) manager node if a jamming attack is mounted at the (+2 −2) zone, as shown in Figure 4.

We can enforce the RDSR in the security view with our DMP. After detecting a jamming attack and determining the detour nodes, each detour node detours the traffic destined for the base station to forward nodes determined according to the process shown in Table 2.

In the basic DMP, we assume a rectangular shaped zone for detouring normal traffic, however our method of defense can be easily adjusted to zones with other shapes such as hexagonal zones or arbitrary shaped zones, because the forward zones are determined among the adjacent neighbor zones. With respect to the zone shape, only the set of neighbor zones differs.

Thus, for the extension of RDSR, we assume each sector of each quadrant is divided into two zones. On the network shown in Figure 4, the candidate forward zones for zone 0 become the neighbor zones 1, 4 and 5 except for jamming zones 2 and 3. Figure 4 depicts the routes by the extension of RDSR with DMP as an illustration of jamming. The *start node* with traffic decides the forward zones among the neighbor zones and then it detours the traffic to the forward nodes in the decided forward zones 1 and 5, ignoring the original RDSR on the boundary of the jamming area. This process is repeated until the general next-hop of a forward node is not a node in the jamming area. After the route moves beyond the jamming area, the remaining routing follows the original RDSR.

## 4. Simulation Results

To evaluate the effectiveness of our scheme, we performed simulations with the GloMoSim (Global Mobile Information Systems Simulation Library) simulator [28] under various parameters. We configured a grid sensor network with 100 nodes, and the average number of one-hop neighbors per node is eight. A sink node is located at a corner of the network and the normal traffic from the sensor nodes is destined for the sink node. As comparative routing mechanisms, static routing with the shortest path, AODV routing [11] and JAM [8] with a detour route are all simulated. As performance metrics, the PDR (Packet Delivery Ratio) and average end-to-end delay of normal traffic are measured. In addition, we figure the ratio of the enhanced PDR and delay in comparison with the JAM approach. For the simulation parameters, we vary the number of normal flows, the number of attack flows, the interval of an attack flow and the number of forward nodes. All jamming traffic is located at the center of network, thus generated normal traffic would be destined for the jamming area in normal routing. All normal traffic is generated by CBR (Constant Bit Rate) traffic at 500 millisecond intervals, and general jamming traffic is generated by CBR traffic at 5 millisecond intervals. In our DMP, the number of multiple paths, **α**, is usually 2. The label “Normal” means that the network has no jamming traffic.

**Figure 5.**
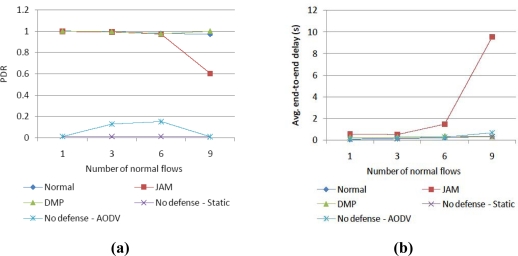
Performance comparison of (a) PDR and (b) delay according to number of normal flows.

In the case of increasing normal traffic, as shown in Figure 5, a dynamic routing protocol, AODV, does not provide fast route recovery against jamming, thus the PDR is very low. The reason that AODV has a low delay is that most normal traffic is blocked in the jamming area and only a little traffic is fast transmitted. JAM provides a high PDR to a degree, but the PDR decreases in the case with a lot of normal traffic and this induces a high delay. On the contrary, DMP provides a high PDR and a low delay that is almost the same as Normal. Through this simulation, the results definitely show that a jamming evasion with a detour route is not sufficient, especially with a lot of normal traffic.

**Figure 6.**
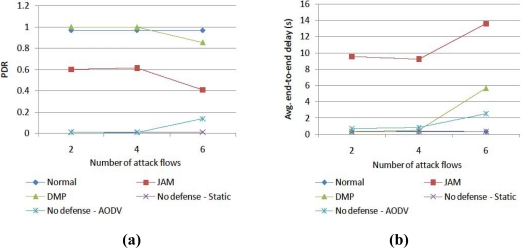
Performance comparison of (a) PDR and (b) delay according to number of attack flows.

Second, we increase the amount of attack traffic as shown in Figure 6, that is, the size of the jamming area. Two, four, and six attack traffic flows produce a jamming effect on 10, 15 and 22 nodes, respectively. In this case also, both AODV and JAM flounder. DMP somewhat worsens the PDR and delay in the big jamming area, because of the long detour routes, but it provides the best performances.

**Figure 7.**
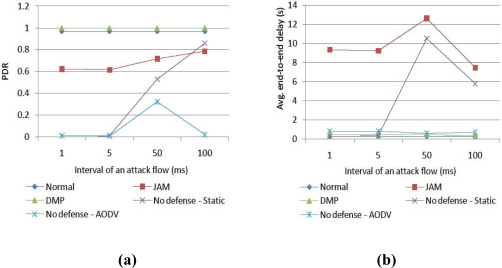
Performance comparison of (a) PDR and (b) delay according to interval of an attack flow.

Third, we vary the interval of the attack traffic with nine normal flows, as shown in Figure 7. Jamming with 1 millisecond is the severest attack. When the strength of jamming is reduced, static routing with the shortest path increases the PDR, but it induces a high delay because the routes pass through the jamming area. JAM enhances the PDR in weak jamming, but it still has a high delay. DMP enhances the PDR more than the normal situation, due to distributed routing with multiple paths.

**Figure 8.**
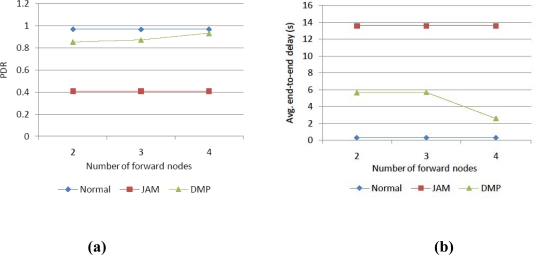
Performance comparison of (a) PDR and (b) delay according to number of forward nodes.

Lastly, we change the number of forward nodes at a detour node in the big jamming area, which has six attack flows. Figure 8 shows that the more forward nodes are determined, the better the performance of DMP in case that jamming is severe.

**Figure 9.**
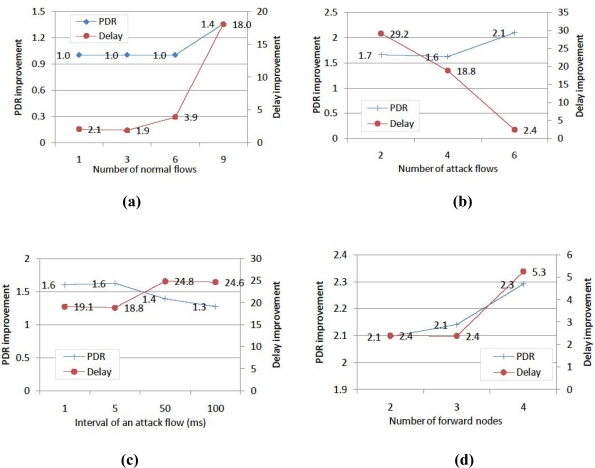
The performance improvement of DMP compared to JAM, according to (a) number of normal flows, (b) number of attack flows, (c) interval of an attack flow and (d) number of forward nodes.

Figure 9 depicts the performance improvement of DMP in comparison with JAM. In all cases, DMP enhances the performance more than JAM and the performance improvement in the PDR is largest in the case with many forward nodes. In particular, the performance improvement in the delay is large, for example it is about 30 times better.

## 5. Conclusions

In order to defend against the jamming attack, one of the most threatening attacks on a ubiquitous networking system, we have designed an immediate detouring service with multiple paths at the only boundary of the jamming area. The simulation results showed that our service enhanced considerably the PDS and average end-to-end delay of normal traffic in comparison with AODV routing and JAM routing. This service will minimize the influence of jamming attacks on real ubiquitous networking systems, such as home automation or industry control systems.

## Figures and Tables

**Table 1.** Comparison of DMP with relative evasion approaches.

**Type**	**Layer**	**Characteristics**

Spectrum Spreading [18,19,20]	Physical Layer	It is too energy-consuming to be widely deployed in resource-constrained sensors.
Channel Hopping [21,22,23]	Link Layer	The jammer can also change the jamming channel continuously and then it enlarges the channel switching overhead on nodes on the entire network.
Retreat [24]	Network Layer	It is limit to mobile environment.
JAM [8]	Network Layer	It simply focused on a mapping service for the jamming area, thus the best single route detouring the jamming zone can easily become congested again.
DMP	Network Layer	As a general approach irrelative with specific protocols on physical and link layer, it can enhance the robustness against jamming on existing routing protocols.

**Table 2.** Algorithm selecting forward nodes at detour node.

1:	**IF** (***n_next-hop_*** ∈ ***N_jamming_***)
2:	***Z_forward_* = *N_forward_*** = **Φ**
3:	**FOR**(each ***z*** in ***Z_neighbor_***)
4:	**IF**((***fn*** in ***z*** ∉ ***N_jamming_***) AND (**c*z_Dist_*** >= ***z_Dist_***) AND (***z*** ≠ ***dz***))
5:	add ***z*** to ***Z_forward_***
6:	**ENDIF**
7:	**ENDFOR**
8:	sort ***fn in Z_forward_*** according to the distance value
9:	
10:	**WHILE**(|***N_forward_***| == **α**)
11:	pop the first node ***fn*** in the sorted list
12:	add ***fn*** to ***N_forward_***
13:	**ENDWHILE**
14:	detour normal traffic evenly to ***fn*** in ***N_forward_***
15:	
16:	**ELSE IF** (***n_next-hop_*** ∉ ***N_jamming_***)
17:	route the traffic according to the general routing protocol
18:	**ENDIF**

***N_jamming_* :** Set of nodes in jamming area

***N_forward_*** : Set of forward nodes

***Z_neighbor_***: Set of neighbor zones

***Z_forward_*** : Set of forward zones

***n_next-hop_***: Next-hop node in general route

***z*** : A zone

***cz*** : A current zone where the detour node is performing DMP

***dz*** : A zone in which the pervious detour node is included, when the traffic is forwarded to the current detour node

***fn*** : A forward node

***z_Dist_*** : Distance of zone ***z*** from the destination zone

**α**: The number of forward nodes given as a system parameter. The detour nodes evenly forward normal traffic on the determined α forward nodes.

## References

[1] What Is Ubiquitous?.

[2] Wood A.D., Stankovic J.A. (2002). Denial of Service in Sensor Networks. Computer.

[3] Li M., Koutsopoulos I., Poovendran R. Optimal jamming attacks and network defense policies in wireless sensor networks.

[4] Raymond D., Marchany R., Brownfield M., Midkiff S. Effects of Denial of Sleep Attacks on Wireless Sensor Network MAC Protocols.

[5] Raymond D.R., Midkiff S.F. (2008). Denial-of-Service in Wireless Sensor Networks: Attacks and Defenses. IEEE Pervasive Comput.

[6] Intanagonwiwat C., Govindan R., Estrin D. Directed diffusion: A scalable and robust communication paradigm for sensor networks.

[7] Kulik J., Heinzelman W.R., Balakrishnan H. (2002). Negotiation-based protocols for disseminating information in wireless sensor networks. Wirel. Netw.

[8] Wood A., Stankovic J., Son S. JAM: A Jammed-Area Mapping Service for Sensor Networks.

[9] Keromytis A., Misra V., Rubenstein D. (2004). SOS: An Architecture for Mitigating DDoS Attacks. IEEE J. Sel. Area. Commun. (JSAC).

[10] Yang H., Luo H., Yang Y., Lu S., Zhang L. HOURS: Achieving DoS Resilience in an Open Service Hierarchy.

[11] Perkins C., Belding-Royer E., Das S. (2003). Ad hoc On-Demand Distance Vector (AODV) Routing. IETF RFC.

[12] Kang B, Ko I. (2010). Effective Route Maintenance and Restoration Schemes in Mobile *Ad Hoc* Networks. Sensors.

[13] Liu M, Cao J., Chen G., Wang X. (2009). An Energy-Aware Routing Protocol in Wireless Sensor Networks. Sensors.

[14] Tufail A, Khayam S.A., Raza M.T., Ali A., Kim K. (2010). An Enhanced Backbone-Assisted Reliable Framework for Wireless Sensor Networks. Sensors.

[15] Al-Karaki J.N., Ul-Mustafa R., Kamal A.E. Data Aggregation in Wireless Sensor Networks - Exact and Approximate Algorithms.

[16] Xu W., Trappe W., Zhang Y., Wood T. The Feasibility of Launching and Detecting Jamming Attacks in Wireless Networks.

[17] Xu W., Ma K., Trappe W., Zhang Y. (2006). Jamming Sensor Networks: Attack and Defense Strategies. IEEE Network.

[18] Chiang J., Hu Y. Cross-layer jamming detection and mitigation in wireless broadcast networks.

[19] Chiang J., Hu Y. Dynamic jamming mitigation for wireless broadcast networks.

[20] Strasser M., Pöpper C., Capkun S., Cagalj M. Jamming-resistant Key Establishment using Uncoordinated Frequency Hopping.

[21] Wood A.D., Stankovic J.A., Zhou G. DEEJAM: Defeating energy-efficient jamming in IEEE 802.15.4-based wireless networks.

[22] Xu W., Trappe W., Zhang Y. Channel surfing: defending wireless sensor networks from interference.

[23] Khattab S., Mosse D., Melhem R. Jamming Mitigation in Multi-Radio Wireless Networks: Reactive or Proactive?.

[24] Ma K., Zhang Y., Trappe W. Mobile Network Management and Robust Spatial Retreats Via Network Dynamics.

[25] Tague P., Li M., Poovendran R. Probabilistic Mitigation of Control Channel Jamming via Random Key Distribution.

[26] Lazos L., Liu S., Krunz M. Mitigating control-channel jamming attacks in multi-channel ad hoc networks.

[27] Oh H., Bahn H., Chae K. (2005). An Energy-Efficient Sensor Routing Scheme for Home Automation Networks. IEEE Trans. Consum. Electron.

[28] GloMoSim.

